# PROTOCOL: The efficacy of nutritional interventions in reducing childhood/youth aggressive and antisocial behavior: A systematic review and meta‐analysis

**DOI:** 10.1002/cl2.1400

**Published:** 2024-04-18

**Authors:** Barna Konkolÿ Thege, Eden Kinzel, Jamie Hartmann‐Boyce, Olivia Choy

**Affiliations:** ^1^ Waypoint Centre for Mental Health Care Waypoint Research Institute Penetanguishene Canada; ^2^ Department of Psychiatry University of Toronto, Temerty Faculty of Medicine Toronto Canada; ^3^ Gerstein Science Information Centre University of Toronto Toronto Canada; ^4^ Department of Health Policy and Promotion University of Massachusetts Amherst Amherst USA; ^5^ School of Social Sciences Nanyang Technological University Singapore Singapore

## Abstract

This is a protocol for a Campbell systematic review of intervention effectiveness. The goal of this systematic review is to answer the following questions based on the available empirical evidence: Are there nutritional interventions (dietary manipulation, fortification or supplementation) that can reduce excessive aggression towards others in children/youth? If yes, how strong is their effect and is there a difference among the three intervention types? Are there nutritional interventions that can reduce antisocial behaviors in children/youth? If yes, how strong is their effect and is there a difference among the intervention types? Are there nutritional interventions that can reduce violent offending in children/youth? If yes, how strong is their effect and is there a difference among the intervention types? Are there nutritional interventions that can reduce non‐violent offending in children/youth? If yes, how strong is their effect and is there a difference among the intervention types? What implementation barriers and solutions to these exist in relation to the above nutritional interventions in children/youth?

## 1 BACKGROUND

### 1.1 The problem, condition, or issue

Aggressive behaviors are common in children and youth and may, in some cases, be developmentally appropriate. However, above‐normal levels of aggressive behavior may result in impairments in family, social or academic functioning, may have acute safety risks and lead to long‐term negative consequences both in the internalizing (e.g., depression) and externalizing problem domains (e.g., antisocial or delinquent behavior resulting in incarceration) (Adesanya, Johnson, & Galanter, 2021; Cleverley, Szatmari, Vaillancourt, Boyle, & Lipman, 2012). Individuals with long‐term, excessive levels of aggression as children and young adults are reported to have a wide range of difficulties including more rule‐breaking behaviors, substance abuse, marital problems, and lower educational as well as occupational attainment (Huesmann, Dubow, & Boxer, 2009). Other data reveal that persistent aggressive behaviors in minors are associated with early sexual activity, early pregnancy, school dropout, and unemployment (Bradshaw, Schaeffer, Petras, & Ialongo, 2010).

When aggressive behavior becomes severe and persistent, it can also be the manifestation or correlate of a psychiatric disorder, such as antisocial personality disorder, oppositional defiant disorder, conduct disorder, attention deficit hyperactivity disorder, autism spectrum disorder, trauma‐related disorders, and others (Ford, Chapman, Connor, & Cruise, 2012).

First‐line treatment recommendations for the treatment of excessive aggression in children and youth include psychosocial interventions according to clinical practice guidelines (T‐MAY guidelines) (Rosato, Correll, Pappadopulos, Chait, Crystal, & Jensen, 2012). These interventions include parent training [supported by the strongest evidence base and largest effect size (Chorpita et al., 2011; Pietro, Valentina, Azzurra, Laura, & Furio, 2018)] as well as cognitive and cognitive‐behavioral approaches (Lee & DiGiuseppe, 2018), among others. Such psychosocial interventions, however, are often suboptimally accessible (e.g., due to costs or the unavailability of professionals to deliver them). Pharmacotherapy may be considered once psychosocial interventions have been shown to be inadequate or unfeasible; treatment guidelines support medications from a variety of classes to treat excessive aggression in children and youth (Gorman et al., 2015). However, the side effect burden is significant and many of these pharmacotherapeutical options are largely not recommended by some more recent guidelines (Gorman et al., 2015). As such, several limitations exist among available psychosocial and psychopharmacological treatment options regarding excessive aggression in terms of access, safety, efficacy, as well as patient and family preferences (Barzman & Findling, 2008; Magalotti, Neudecker, Zaraa, & McVoy, 2019; Pisano & Masi, 2020).

### 1.2 The intervention

An individual‐level nutritional intervention is a set of actions designed to improve the nutritional status of the individual using one of the following three activities: (1) dietary manipulation, which aims to modify the individual's natural eating habits (e.g., consuming more food naturally rich in Vitamin D); (2) fortification, which is the addition of nutrients to the basic foods the individual consumes (e.g., drinking milk with added Vitamin D), and (3) supplementation, which entails administering a specific (set of) nutrient(s) separate from the components of the individual's default diet (e.g., taking a Vitamin D supplement) (Martínez‐López, Pérez‐Guerrero, Torres‐Carrillo, López‐Quintero, Betancourt‐Núñez, & Gutiérrez‐Hurtado, 2022).

While dietary manipulation should be the first choice when trying to improve the nutritional status of individuals, such attempts often fail due to a variety of reasons. It is well‐documented, for example, that difficulties with adherence to dietary modifications hinders the effectiveness of such interventions (World Health Organization, 2003). Further, above‐average nutritional needs due to inherited metabolic characteristics, chronic stress, use of certain medications, or poor gut health as well as reduced nutrient content of natural food sources are all potential factors that can prevent dietary modifications reaching their intended purpose (Rucklidge, Johnstone, & Kaplan, 2021).

Nutritional supplements are widely accepted and commonly used. According to a Canadian general population survey, 46.9% of women and 33.5% of men reported taking at least one nutritional supplement in the month preceding the survey (Vatanparast, Adolphe, & Whiting, 2010). The same values in a study conducted in the United States were 53% and 44%, respectively (Bailey et al., 2010), while yet another study reported that 33% of children and adolescents used dietary supplements in 2013–2014 (Qato, Alexander, Guadamuz, & Lindau, 2018). Supplementation is also common with the specific purpose of alleviating certain mental health disorder symptoms [e.g., 31%–33% for attention deficit hyperactivity disorder (Chan, Rappaport, & Kemper, 2003; Gardiner, Buettner, Davis, Phillips, & Kemper, 2008)].

In addition, nutritional interventions can also be relevant to a criminal justice‐involved population because the prevalence of nutritional deficiency in incarcerated samples is high. For example, one recent meta‐analysis estimated the prevalence of vitamin D deficiency in individuals in prison worldwide to be 55% (Tripathy, Negi, Kumar, & Shamim, 2023). This finding may be attributed not only to challenges incarcerated individuals face in accessing healthy diets (Smoyer, 2019), but also to food choices people who have offended make that undermine the nutritional balance of institutionally provided meals. This is evidenced by the finding in imprisoned young people in the UK where despite being given a diet with nutrient contents close to recommended guidelines, the intakes of some vitamins and minerals, such as vitamin D and selenium, fell below recommendations (Eves & Gesch, 2003). Failure to achieve recommended nutrient intakes in criminal justice facilities has similarly been documented in other countries such as Australia and the USA (e.g., Hannan‐Jones & Capra, 2016; Jacobs & Mullany, 2015). These findings suggest that it would be apt to consider the impact of dietary modifications in youths in these institutional settings. Furthermore, the applicability of nutritional interventions to a criminal justice‐involved population of youths is strengthened by the fact that unlike individuals in other institutional settings, prisoners are entirely dependent on food in the prison for their nutritional well‐being. Such interventions also align with overarching aims to promote health within prison contexts, especially in the UK (Woodall, 2016).

### 1.3 How the intervention might work

The potential of nutrients such as vitamins, minerals, and amino acids to reduce aggression and violence is an active area of study within the field of nutritional psychiatry (Rucklidge, Kaplan, & Mulder, 2015). A body of literature documents links between nutritional status and a variety of antisocial behaviors. In prospective longitudinal studies of children and young adults, there is evidence that children with malnutrition in the first years of life exhibited more conduct problems in adolescence (e.g., Galler et al., 2012; Liu, Raine, Venables, & Mednick, 2004). Moreover, a higher intake of processed foods, red meat, high‐fat dairy products, and high‐sugar foods at age 11 years was associated with increased externalizing behaviors at age 14 in a large prospective study of Australian youth (Trapp et al., 2016). Another longitudinal study from a lower‐income country (Colombia) showed that a diet richer in dairy products and higher quality meat—in contrast to diets rich in carbohydrates or lower quality meat without dairy products—predicted lower level of aggression 5–9 years later even when controlling for numerous sociodemographic confounders (Robinson, Mora‐Plazas, Oliveros, Marin, Lozoff, & Villamor, 2021). A large, representative study of Brazilian students revealed a significant association between unhealthy diet (more processed food and refined carbohydrates, while less fruits, vegetables, and legumes) and bullying perpetration, including sexual harassment and physical aggression, again after controlling for numerous sociodemographic confounders (Okada et al., 2024).

Another strand of evidence for the association between nutritional status and offending stems from research on food insecurity. In an assessment of delinquent behaviors that included not only aggressive acts but also non‐aggressive ones such substance use, truancy, and vandalism, children who were raised in households with limited or uncertain abilities to acquire nutritionally adequate foods exhibited higher levels of delinquency (Jackson, Newsome, Vaughn, & Johnson, 2018). The link between poor nutritional status and antisocial behavior may be exacerbated by the fact that food preferences and choices are rooted in factors such as socioeconomic status that are risk factors for offending. Additional support for the nutrition‐antisocial behavior relationship comes from evidence that improvements in early childhood nutrition can lead to reductions in offending at age 24 years, with each additional year of a nutritional assistance program reducing the likelihood of a criminal conviction in young adulthood by 2.5% (Barr & Smith, 2023). Notably, the significant and larger reductions in convictions following nutritional assistance were observed for violent crimes, but not for property crime (Barr & Smith, 2023). While the above data do not provide definitive evidence for a causal relationship due to their observational nature, they point in the direction that certain dietary patterns may lead to an increase in the occurrence of antisocial/violent behaviors and consequently, it could be that improving nutritional status may reduce aggressive behaviors.

The neurobiology of excessive aggression is complex and poorly understood. Neurochemical systems can impact aggression in at least two ways: by influencing central nervous system development during critical periods and modulating neuronal functioning of the already developed nervous system throughout life (Rosell & Siever, 2015). Both the serotonin and dopamine systems have been shown to play a role in modulating aggression in addition to GABA, oxytocin, testosterone and cortisol (Siever, 2008). Several important gene‐by‐environment interactions also exist (Siever, 2008), including specific genetic polymorphisms and early adversity that in combination may predispose to aggression (Rosell & Siever, 2015).

Adaptive aggression regulation is dependent on both the healthy development of the nervous system in childhood and adolescence as well as an ongoing balance between bottom‐up subcortical processes and top‐down cortical modulation (Siever, 2008). All of these processes are reliant on—among other factors—the adequate level of nutrients available in the body to support the development and optimal functioning of the central nervous system (Roberts, Tolar‐Peterson, Reynolds, Wall, Reeder, & Rico Mendez, 2022). For instance, omega‐3 fatty acids are necessary for general neurodevelopment as they are building blocks of brain cell membranes including neuronal synapses (Gajos & Beaver, 2016).

**Figure 1:**
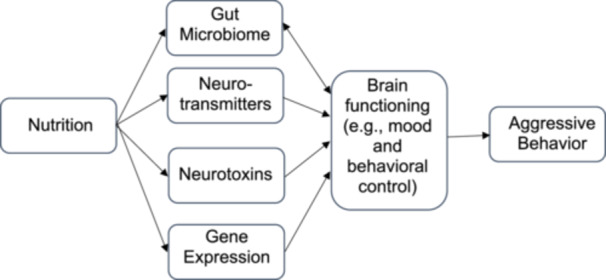
Proposed mechanisms underlying the association between nutritional status and aggressive/antisocial behavior.

In addition to playing a more direct role in brain cell growth and development, there are several proposed mechanisms by which nutrients may affect brain function to in turn influence behavior (Figure 1). First, micronutrients (vitamins and minerals) serve as co‐factors in the synthesis of neurotransmitters (e.g., serotonin, dopamine, GABA) (Rucklidge, Johnstone, & Kaplan, 2021). Other nutritional factors such as omega‐3 fatty acid deficiency can also lead to dysfunctional serotonin synthesis, activation, and function (Patrick & Ames, 2015), while a high protein intake can impact the metabolism of tyrosine which is a precursor of dopamine (Muth & Park, 2021), highlighting the effect of nutrition on changes in neurochemistry. Second, as nutrition is an important modulator of toxicity from environmental chemicals, malnutrition can affect the brain by exacerbating neurotoxins, while intake of nutrients associated with neuroprotective effects can help to retain brain structural integrity (Muth & Park, 2021). Third, micronutrients are also involved in the methylation‐folate cycle which can affect genetic expression (Rucklidge, Johnstone, & Kaplan, 2021). A fourth mechanism involves the gut microbiome, which is also influenced by diet. Signals sent along the gut‐brain axis make their way to the brain to regulate behavior (Dinan, Stilling, Stanton, & Cryan, 2015; Tcherni‐Buzzeo, 2023). Although empirical research on the gut‐brain axis and human aggression is currently lacking, this pathway from microbiome to aggression has been proposed based on evidence of the association of the microbiome with mental health outcomes and psychological factors related to aggressive behavior. Some significant differences in the gut microbiota were found between children and adolescents with attention‐deficit/hyperactivity disorder and control groups (Soltysova, Tomova, & Ostatnikova, 2022), while gut microbial structure was found to be associated with temperament in young children (Christian, Galley, Hade, Schoppe‐Sullivan, Kamp Dush, & Bailey, 2015). Consequently, adaptive aggression regulation is also dependent on nutritional status, which if suboptimal, can be improved by nutritional interventions.

### 1.4 Why it is important to do this review

Among the interventions that have the potential to decrease aggression in children and youth, many are relatively difficult to access (e.g., require highly trained professionals for whom the demand highly exceeds supply) or punitively expensive either to service users directly or society as a whole. In contrast, nutritional interventions cost less [e.g., nutritional supplements versus psychiatric medications (Kaplan, Isaranuwatchai, & Hoch, 2017)] or nothing (e.g., elimination diets) and so are generally more accessible. If the synthesis of the evidence can confirm the effectiveness of (certain) nutritional interventions in reducing aggression/violence in children and youth, stakeholders and policy makers will have tools, which are readily employable potentially even on larger scales given current Western societies’ high level of interest in healthy nutrition.

It is also worthy of mentioning that many dietary interventions investigated to reduce aggression are not just helpful in reducing aggression in an isolated manner: instead, they often have a broader beneficial effect on mental health (Kaplan & Rucklidge, 2021) given the importance of healthier nutrition in relation to (brain) health in general.

The present review is needed as prior reviews on this topic are vastly outdated (Benton, 2007), specific to a single nutritional intervention (Gajos & Beaver, 2016; Hibbeln & Gow, 2014) or a subgroup of them (Rucklidge & Kaplan, 2013) or do not contain quantitative synthesis of the data (Qamar, Wang, Qureshi, LaChance, Kolla, & Konkolÿ Thege, 2023; Qureshi, Kunaratnam, Kolla, & Konkolÿ Thege, 2021) or focus on supplementation but not dietary modifications. They also did not cover a broader conceptualization of problem behaviors (cf. additional focus on antisocial behaviors in the present review) but other‐directed aggression per se only. Finally, prior reviews do not explore implementation challenges and potential solutions to these issues. No ongoing review with a comparable focus has been identified by this author team.

## 2 OBJECTIVES

The goal of this systematic review is to answer the following questions based on the available empirical evidence:

- Are there nutritional interventions (dietary manipulation, fortification or supplementation) that can reduce excessive aggression towards others in children/youth? If yes, how strong is their effect and is there a difference among the three intervention types?
- Are there nutritional interventions that can reduce antisocial behaviors in children/youth? If yes, how strong is their effect and is there a difference among the intervention types?
- Are there nutritional interventions that can reduce violent offending in children/youth? If yes, how strong is their effect and is there a difference among the intervention types?
- Are there nutritional interventions that can reduce non‐violent offending in children/youth? If yes, how strong is their effect and is there a difference among the intervention types?
- What implementation barriers and solutions to these exist in relation to the above nutritional interventions in children/youth?

## 3 METHODS

This review will follow the methodological expectations of intervention reviews published by the Campbell Collaboration (Methods Coordinating Group of the Campbell Collaboration, 2019).

### 3.1 Criteria for considering studies for this review

#### 3.1.1 Types of studies

Considering the relatively novel nature of the field of nutritional psychiatry (Adan et al., 2019; T. Dinan, 2023; Marx, Moseley, Berk, & Jacka, 2017; Sarris et al., 2015) and accordingly, the limited amount of data accumulated to date, we are planning to consider any prospective study employing a controlled design (having an intervention and a comparison group) for the purposes of the quantitative evidence synthesis. Consequently, we will not only consider randomized (or quasi‐randomized) controlled trials but quasi‐experimental studies (observational cohort designs) as well.

We will not consider epidemiological studies cross‐sectionally describing the association between nutritional status/dietary patterns and the selected outcome variables as this review will focus on the efficacy of *interventions*. We will not consider studies with simple pretest‐posttest design either due to concerns of internal validity. If our resources will allow, we will consider these latter two types of studies when forming recommendations for future research.

All types of studies (theoretical, quantitative with any designs, qualitative) will be considered in the mixed method synthesis of the present review aiming to support the better understanding of the barriers and facilitators of the successful implementation of nutritional interventions.

#### 3.1.2 Types of participants

This study will focus on children and youth presenting with elevated level of aggression.

From the age perspective, relevant study participants will be defined as individuals up to the age of 24 (regardless of sex/gender). This particular age as the exit from youth is somewhat arbitrary and debatable; however, individuals aged 18–24 are often considered as transitional‐aged youth with characteristics and needs somewhat different than those of minors or adults. Further, in the context of nutrition and brain functioning, the early twenties is the age when maturation of the brain becomes mostly complete (Arain et al., 2013); in contrast to the age of 18, which could be used as an alternative threshold for adulthood but more from the legal than the neurodevelopmental or psychological perspective. If a study includes participants whose age is older than 24 years old and there is no way to separate those at or under this age, the study will be included in the review if the mean (if reported, median if mean not reported) age is 24 years or less. If the mean (or median) age of the sample is more than 24 years of age and we cannot separate out data from those 24 and younger, the study will be excluded even if it contains some participants who would be relevant for this review.

In terms of excessive aggression, it is important to note that certain level of aggression, especially with younger children, can be age‐appropriate. This review intends to consider data in relation to *elevated*/*maladaptive* level of aggression, for which we will seek an indicator (e.g., mental health care utilization for disorders often co‐occurring with aggression such as attention‐deficit/hyperactivity disorder, antisocial personality disorder, oppositional defiant disorder or autism; being in the criminal justice system for whatever reason either in a prison or special educational setting; being characterized by above‐normal self‐rated or observer‐rated scale scores measuring aggression etc.). Studies on participants with no—even indirect as described above—indication of excessive level of aggression (either at baseline or intervention end) will be excluded as no effect is anticipated from a nutritional intervention in relation to normative level of aggression (we anticipate that this situation can occur with the highest likelihood in studies where aggression‐related variables are secondary and therefore excessive level of aggression did not play a role in participant selection).

#### 3.1.3 Types of interventions

We are going to consider two main types of nutritional interventions; namely, dietary manipulation and nutritional supplementation, both of which should be long enough in duration (minimum of one week) so that a significant change in the individual's nutritional status can be expected.

Dietary manipulation is an attempt to intentionally change an individual's natural food consumption patterns to improve nutritional status (i.e., for example a one‐time, high‐sugar drink intake in a laboratory environment to test the negative effects of excess sugar would not be considered as a dietary intervention for the purposes of this review). The aim of such interventions is to either (1) increase the consumption of certain foods rich in nutrients which are not readily available in the individual's system in the required amount; or (2) decrease the consumption of or completely eliminate certain foods containing substances which are (a) necessary and helpful up to a certain amount but harmful in excess (e.g., lipids or carbohydrates) or; (b) unnecessary or directly harmful for the human body (e.g., certain food additives that get into food through industrial food production).

In contrast, nutritional supplementation/fortification exclusively aims to increase the availabilities of nutrients in the individual's system. In the case of nutritional supplements this happens through the consumption of manufactured products (in the form of pills, capsules, tablets, or liquids) that are regulated as dietary supplements (and not medications; thus not requiring prescription by a physician). Both nutritional supplementation and fortification intend to supplement diet with substances that have been confirmed as being essential to life, which can be micronutrients such as vitamins (vitamin A, vitamin B, etc.) and minerals (calcium, magnesium, zinc, etc.) or macronutrients (e.g., fatty or amino acids) or a combination of these. Additionally, phytoceuticals, that is, plant‐based natural products will also be considered. Supplementation is defined as the consumption of manufactured supplements in addition to diet.

In controlled studies on the efficacy of nutritional interventions in relation to behavioral outcomes, the comparators can be quite diverse ranging from placebo (e.g., tablets/capsules with identical outer characteristics as the supplement, or similarly looking and tasting food/drink if the supplementation occurs through the consumption of nutrient‐fortified foods) through different psychosocial interventions to “treatment as usual.” Given the preliminary stage of evidence in nutritional psychiatry, we are planning to consider only “non‐active” comparators (e.g., placebo, treatment as usual) or active comparators as an add‐on to a nutritional intervention (e.g., cognitive therapy combined with a nutritional intervention in comparison with cognitive therapy alone) in the main analyses. We anticipate that we will find only a negligible amount of studies where the comparator will be active (e.g., another nutritional intervention or cognitive therapy); in case of identifying such studies, we will conduct the head‐to‐head comparisons in separate analyses.

#### 3.1.4 Types of outcomes

The primary outcomes in this study will be (1) behavioral‐level violence/aggression toward others (people or objects) in real‐life (non‐simulated) settings; (2) antisocial behaviors; (3) violent offending; and (4) non‐violent offending.

Violent/hetero‐aggressive behavior can be defined as intentionally causing or attempting to cause emotional or physical harm or damage to somebody other than the acting person. For the purposes of this review, violent/aggressive behavior will include both reactive aggression, that is, impulsive violence or threat‐driven aggression, as well as proactive aggression or violence committed with a purpose to increase one's dominance or to obtain property (Fossati et al., 2009). In accordance with the above considerations, studies will be excluded if they investigate: (1) aggressive/angry/hostile emotions or thoughts without observable behaviors; or (2) aggressive tendencies presented in simulated environments (e.g., level of aggression expressed in a video game play situation, which is thought to be qualitatively different from real‐life situations).

As behavioral‐level violence and aggression in children and youth is often not studied in isolation but in addition to or as part of an amalgamation of antisocial behaviors, this more heterogeneous conceptualization—including, for example, disobedience, theft, lying, intentional property damage—will also be considered.

As an extreme form of hetero‐aggressive behavior, violent offending will be considered as a separate outcome due to the high societal importance of its reduction. A violent offense will be defined as actual, attempted, or threatened harm directed toward another person (including nonconsensual sexual contact between the perpetrator and another person) sanctioned by the law of the jurisdiction of the perpetrator.

Finally, due to the important differences in consequences both for the perpetrator and society as a whole, non‐violent offending will also be considered as a separate outcome, independent of both violent offending and non‐sanctioned antisocial behaviors. In cases, where violent and non‐violent crime will be reported in a combined format—but only in that case—we will consider a general outcome of criminal offending including information on both violent and non‐violent crimes.

#### 3.1.5 Outcome measures

Hetero‐aggression is typically operationalized by observer‐rated scales but occasionally self‐reported questionnaires are also used. An example for the former is the Aggression Subscale of the Child Behaviour Checklist (Achenbach & Ruffle, 2000), while examples for the latter include the Aggressive Behavior Scale of the Youth Self Report (Ebesutani, Bernstein, Martinez, Chorpita, & Weisz, 2011), the Buss‐Perry Aggression Questionnaire (Buss & Perry, 1992), and the Reactive‐Proactive Aggression Questionnaire (Raine et al., 2006). Data produced by either of these methods will be considered.

Similar to aggressive behaviors, antisocial behaviors are also typically operationalized by observer‐reported scales or institutional records but occasionally other quantitative approaches (e.g., self‐reported scales) are also used. Examples for the former include the Conduct Subscale of the Strengths and Difficulties Questionnaire (Goodman, 2001), the Irritability Subscale of the Aberrant Behavior Checklist (Aman, Singh, Stewart, & Field, 1985) or records of institutional misconduct, while an example for the latter is the Rule‐breaking Behavior Subscale of the Youth Self‐Report (Ebesutani et al., 2011).

Offending is most often operationalized by some ratio of offending or recidivism as expressed by criminal/legal records. Occasionally self‐reports on the same are also used; both of these methods will be considered in this review.

#### 3.1.6 Duration of follow‐up

If multiple data collection occur during the intervention period, end‐of‐intervention data will be considered only as we assume that the fullest intervention effect will be observable at that point [especially given the typically short intervention duration used in the extant literature; cf. (Qamar et al., 2023)].

According to the hypothesized mechanisms of action of the intervention, the consistent availability of nutrients is needed on a long‐term basis for the healthy development and functioning of the central nervous system. Therefore, we do not anticipate that improved behavioral outcomes could be observed after the discontinuation of the intervention. To investigate this hypothesis, we will also extract follow‐up data from the first time point closest to 3‐month post‐intervention [which is the median duration of interventions with a similar focus; cf. (Qamar et al., 2023)].

#### 3.1.7 Types of settings

While research on aggression and violence is common in clinical populations (i.e., among children and youth with a mental health disorder diagnosed by a mental health care professional), both aggression/violence and antisocial behaviors in general are commonly occurring and frequently studied phenomena in non‐clinical populations as well (e.g., in educational or criminal justice settings). We argue that the non‐clinical setting or the non‐existence of a psychiatric diagnosis is often not an indication of the lack of a disorder but rather the lack of resources to identify and treat those mental disorders—regardless of the setting. Therefore, we are not planning to restrict our interest to any particular setting or diagnostic category but instead, will collect and synthesize the evidence on children and youth in general who present with observable, maladaptive level of aggression or antisocial behaviors.

### 3.2 Search methods for identification of studies

After consulting the Campbell Collaboration's search‐specific methodological guidelines (Kugley et al., 2017), a comprehensive search for published and unpublished studies and reports will be performed to reduce the risk of publication bias and identify the best available evidence. No date or language restriction will be applied when attempting to identify relevant studies; however, the search terms will be used in English only; therefore, only studies with an English language title and/or abstract will be considered as potentially eligible.

#### 3.2.1 Electronic searches

Comprehensive database searches will be designed in collaboration with a health sciences librarian (EAK) and the initial Ovid MEDLINE search will be peer reviewed by another health sciences librarian following the PRESS protocol (McGowan, Sampson, Salzwedel, Cogo, Foerster, & Lefebvre, 2016). The search will be modified slightly for each database but will generally consist of a combination of keyword terms and controlled vocabulary (when available) for each of the following concepts: 1. aggression, violence, antisocial behaviors, and offending; 2. diet, nutrition, vitamins, minerals, nutritional supplements, and dietary interventions; and 3. youth under the age of 25. The complete Ovid MEDLINE search string is provided in Appendix A. This algorithm will be translated and also run in Ovid Embase, Cochrane via Wiley, Ovid PsycINFO, Scopus and the Allied and Complementary Medicine Database.

#### 3.2.2 Searching other resources

The reference list of all relevant systematic or narrative reviews identified through the data base searches will be checked for additional relevant original studies. Further, reference list of each eligible original study will also be checked to identify any additional studies missed by the database search. In addition, the corresponding author of each eligible study and further experts will be contacted to (1) identify unpublished or otherwise missed but relevant original studies and (2) seek out direct information (and sources of such information) on implementation barriers and potential solutions regarding the nutritional interventions studied.

The clinical trials register of the (US) National Library of Medicine (https://clinicaltrials.gov) and the International Clinical Trials Registry Platform (https://www.who.int/clinical-trials-registry-platform) as well as preprint databases (Research Square: https://www.researchsquare.com; medrXiv: medrxiv.org; Open Science Foundation: https://osf.io/preprints/) will be searched for unpublished studies. ProQuest Dissertations & Theses and EBSCO Open Dissertations will be searched for potentially relevant dissertation and thesis work. Google Scholar will also be used (screening to be continued for 50 hits after the last relevant record) to make the searches as comprehensive as possible. Searches in these databases will be conducted using a simplified algorithm employing different combinations of our most central search terms (i.e., nutrition, diet, aggression, violence, antisocial behavior, and offending).

Additionally, we will search for relevant information on the websites of (federal‐level) public health agencies of Australia, Canada, UK, and the USA, the e‐Library of Evidence for Nutrition Actions, the USDA Nutrition Evidence Systematic Review, and the What Works Network. Further, the journal Evidence‐based Complementary and Alternative Medicine will be hand‐searched.

### 3.3 Data collection and analysis

#### 3.3.1 Description of methods used in primary research

We anticipate that the majority of relevant studies will employ a randomized controlled design, however, some studies may use other forms of controlled design (e.g., quasi‐experimental studies). In this latter case, we will assess—as part of the risk of bias assessment process—whether the study groups will be matched on the most important baseline participant characteristics (e.g., mental disorder diagnosis, criminal justice system involvement, age, sex).

#### 3.3.2 Selection of studies

After completing the searches, all identified citations will be collated and uploaded into the EndNote 21 reference management system (https://endnote.com). After removing duplicates by Endnote, records will be uploaded to the Covidence systematic review platform (https://www.covidence.org). Titles and abstracts will be screened by two independent evaluators (L. M., C. R.). The full text of all records that were deemed potentially relevant by either reviewer will be retrieved and checked for inclusion and exclusion criteria by both reviewers. Discrepancies between reviewers in terms of eligibility assessments will be resolved by consulting a third reviewer, the senior author of the study (B. K. T.).

Decision on the inclusion of seemingly eligible studies (based on title and abstract) written in a language other than English will be made at this stage—considering the time and financial resources of the review team available at that point to arrange reliable translation. Studies excluded due to language barriers will be collated and referenced in the final review to allow knowledge users with more resources to consider those reports.

#### 3.3.3 Data extraction and management

Studies satisfying eligibility criteria will be split in half, with one coder (L. M./C. R.) conducting primary data extraction for each study in their set. A third coder, the senior author of the review (B. K. T.), will independently validate all coding for all studies by cross‐checking the extracted data with the original research report. Discrepancies between each pair of coders will be discussed with the inclusion of the third coder until consensus is reached.

A coding sheet will be piloted on at least 10 studies and revised if needed. The draft coding sheet can be found in the supplementary materials to this article. The actual data extraction process will be managed in the Covidence systematic review platform (https://www.covidence.org).

#### 3.3.4 Assessment of risk of bias in included studies

Studies’ potential risk of bias will be assessed by two independent reviewers (those who have completed the data extraction for the given study). All studies will be scored across several domains resulting in an overall rating of high, medium, or low risk of bias using the appropriate version (i.e., developed for parallel, cross‐over or cluster‐randomized trials) of the revised Cochrane Risk of Bias tool for randomized studies [RoB‐2; (Eldridge et al., 2021; Higgins, Li, & Sterne, 2021; Higgins, Savović, Page, & Sterne, 2019)] and the Cochrane Risk of Bias in Non‐randomized Studies of Interventions [ROBINS‐I; (Sterne et al., 2016)].

#### 3.3.5 Measures of treatment effect

To perform the meta‐analysis, data from each original report will be standardized so that results across studies can be meaningfully combined.

For continuous outcomes (e.g., total scores on an observer‐rated scale measuring aggression toward others), Hedges’ *g* will be computed. This metric is preferred over Cohen's *d* for small samples, which is expected to be the case for many studies to be included in this review. The main source of information needed here will be means and standard deviations but in situations where these are not provided, alternative sources of information (e.g., *f*‐values, *p*‐values) will be used. For dichotomous outcome data (e.g., raw numbers or proportions representing how many participants committed an offense), effect sizes will be computed as odds ratios.

#### 3.3.6 Unit of analysis issues

If a study includes more than two study arms, no subgroup will be considered twice in the same meta‐analysis (including the control group). If a study arm combines a nutritional and another type of intervention, this arm will be considered only if there is another arm where the non‐nutritional intervention is studied separately, and this latter will be used as the control group (even if there is a no‐intervention control group, which rather will be used as a comparator for the study arm where the nutritional one is the only intervention).

Repeated observation on participants will be handled according to the principles laid out in the section “Duration of follow‐up.”

To account for the effects of clustering in the case of cluster‐randomized trials, necessary adjustments to the analyses will be made using intra‐cluster correlation described in the work of (Hedges, 2007). If the intra‐cluster correlation coefficient is not available, we will use values from similar studies conducted on a similar population or will extract it from extant reviews.

In case of cross‐over studies, we will only combine the data with parallel‐group randomized controlled trials if outcome data are available from the first phase of the study separately (up to the point of cross‐over). If such data are not available, we will analyze cross‐over studies in analyses separate from that of the parallel‐group randomized controlled trials.

#### 3.3.7 Criteria for determination of independent findings

The primary unit of the analysis for the quantitative investigation will be the individual (and not the study); therefore, if several papers report on the same study involving the same participants, the data will be treated as a single case for the purposes of this review and the data point with the most comprehensive description will be considered.

For every outcome of interest (i.e., aggression towards others, antisocial behaviors, violent offending, and non‐violent offending) a single effect size will be extracted for each eligible study. If multiple effect sizes will be presented in the same study for the same outcome, the following decision rules (López‐López, Page, Lipsey, & Higgins, 2018) will be applied:

1. Observer‐reported outcomes will be prioritized over self‐reported outcomes due to the risk of social desirability effect with self‐reported data regarding aggression.
2. If data on reactive and proactive aggression will be provided separately, an aggregated effect size based on both indicators will be calculated given the equal relevance of both constructs for the purposes of this review. However, we will test whether the type of aggression (reactive or proactive) moderates any treatment effect by also calculating effect sizes for reactive and proactive aggression in separate analyses.
3. More comprehensive conceptualizations or measurements of aggression will be prioritized over sub‐scores (e.g., a total scale score will be prioritized over a subscale score if all subscales measure different aspects of the same outcome; a single item encompassing aggression in general over another item that only covers a subarea of aggression).
4. If only sub‐scale scores are reported, more severe forms of aggression will be prioritized over less severe forms of aggression (e.g., physical violence over verbal threats).
5. If data for similar composite indicators of aggression will be reported, we will prioritize the one which does not include information on auto‐aggression (as we argue that auto‐aggression is a qualitatively different construct from aggression directed toward other people or property).
6. Data based on assessment tools used more often in the included studies will be prioritized over ad hoc or only rarely used measures to increase comparability.
7. If multiple interventions are examined within the same study while measuring the same outcome, and the only difference between the intervention variants will be in dosage, we will prioritize the intervention with the dosage that is closer the to the current Recommended Dietary Allowances of the given nutrient(s) according to the U.S. Food and Drug Administration (Food and Drug Administration, 2016).

If even after considering these principles, no clear priority can be established, the outcome to include in the given meta‐analysis will be selected randomly from those outcomes satisfying the above criteria, and sensitivity analyses will be conducted testing the impact of outcome choice on the overall pooled estimate (see below). All non‐selected but relevant outcomes or study arms (e.g., different dosages of the same nutritional supplement) from each eligible study will be listed and reported to facilitate later, more nuanced analyses using correlated‐hierarchical effect models to appropriately study dependent data (which will not be part of this study due to resource limitations).

#### 3.3.8 Dealing with missing data

If the required data (e.g., means and standard deviations) are not available in the published original reports, we will use alternative raw data available within the included studies (e.g., *p* value from *t*‐test) to generate an effect size metric. All required data transformation will be conducted using the web‐based calculator developed by Wilson (Wilson, 2001). If no data are available in the original reports that would allow the calculation of the pooled effect size, the authors of the original reports will be contacted and the data will be requested. Should such an attempt fail, the given original study will be excluded from the quantitative analysis and will be reported on in the narrative synthesis only.

#### 3.3.9 Assessment of heterogeneity

Heterogeneity amongst original studies will be assessed using the *τ* ^2^ and *I* ^2^ statistics (Higgins, Thompson, Deeks, & Altman, 2003). An *I* ^2^ value between 30% and 50% will be considered as a sign of moderate heterogeneity, while values between 50% and 90% will be considered as an indication of substantial heterogeneity. We will also calculate and report 95% prediction intervals to quantify heterogeneity.

#### 3.3.10 Assessment of reporting biases

We will use funnel plots for information about possible publication bias if there are 10 or more studies included in a given meta‐analysis (Sterne et al., 2011). Statistical examination of funnel plot asymmetry (Egger test) will also be performed. If this test will confirm the existence of funnel plot asymmetry, we will attempt to explain possible sources of the identified reporting bias keeping in mind that asymmetric funnel plots are not necessarily caused by publication bias.

#### 3.3.11 Data synthesis

Studies will be grouped according to outcomes (i.e., aggression toward other people or property, antisocial behavior, violent and non‐violent offending) and potentially intervention characteristics (see more details in this regard in the section “Subgroup analysis”). Where the outcome‐specific meta‐analysis will combine data based on different scales or measures, we will ensure that higher scores or odds indicate higher level of aggression.

We are planning to use RevMan Web for the statistical analyses as a default but if that software would not be able to handle certain type of data or conduct certain analyses, SPSS 28 or Comprehensive Meta‐Analysis V4 will be used. The random effect model will be used and beyond the point estimates, 95% confidence intervals will be calculated. A graphical representation of the results (forest plot) will also be provided for each outcome separately.

Where meta‐analysis is not feasible, findings will be presented in narrative form including tables to aid in data presentation.

#### 3.3.12 Subgroup analysis and investigation of heterogeneity

Subgroup analyses will be considered for each separate outcome variable depending on the number of studies per outcome. If applicable, subgroups will be formed based on:

1) intervention type (e.g., diet change versus nutritional supplementation; single‐ versus broad‐spectrum nutritional supplementation);
2) intervention duration [less or more than 15 weeks, which was the mean intervention duration in a review with a similar focus (Qamar et al., 2023)];
3) study design (parallel‐group randomized controlled, cross‐over, versus observational cohort designs);
4) study population (e.g., health care, criminal justice setting, or other);
5) and sociodemographic characteristics of participants (age, sex, and ethnicity).

#### 3.3.13 Sensitivity analysis

The generalizability and robustness of the results will be examined by sensitivity analyses conducted after removing studies characterized by high risk of bias. As noted above, where a study reports multiple outcome measures and we cannot establish superiority based on our pre‐defined criteria, we will also test the sensitivity of pooled estimates to the inclusion of other relevant outcome measures.

#### 3.3.14 Treatment of qualitative research and mixed method analysis

Considering the need for practical usability of the findings, beyond the standard quantitative data collection and synthesis on intervention efficacy, we are also planning to collect and summarize data on implementation barriers and facilitators. For this purpose, we will consider non‐empirical (theoretical) as well as quantitative and qualitative empirical data. We will organize these data using a causal chain analysis (White, 2018); that is, we will consider the different stages of implementation and will seek for and organize implementation‐related information corresponding to these stages (Table 1).

**Table 1:** Stages of the causal chain with information to be considered at each stage.

Stage in causal chain	Data
Awareness of the relevance of and interest in the intervention among relevant decision makers and the target group	Level of awareness for example in individuals with high risk for committing violent/antisocial acts, in parents or physicians of children with conduct problems or in officers of the criminal justice system that nutrition is relevant to prevent or manage aggression and conduct problems. Motivation in the same groups regarding seeking out specific information on what type of nutritional intervention would be most relevant/feasible considering the specific circumstances (cf. changing a family's vs. a prison's dietary patterns).
Access to the nutritional intervention	Accessibility of nutritional supplements or main elements of a healthy diet. Financial costs associated with nutritional supplements or main elements of a healthy diet.
Characteristics of nutritional interventions	Nutrients targeted by interventions, dosage, and length of interventions.
Compliance with the nutritional intervention	Compliance with versus drop‐out rates from nutritional interventions. Factors contributing to compliance versus drop‐outs including side effect burden.
Treatment‐interfering behaviors or physiological processes	Factors influencing the effectiveness of successfully implemented nutritional interventions (e.g., above‐expected nutritional needs due to illness, medications or idiosyncratic metabolic needs, gut health).

We assume for example, that the potential benefits of nutritional interventions in reducing aggression and violence is often not just unknown but unimaginable to many decision makers (whether parents or directors of criminal justice facilities) making lack of knowledge a major barrier towards the implementation of such interventions. Similarly, compliance with any nutritional intervention can be challenging to many intervention recipients or their social environment due to the efforts required (e.g., replacing well‐liked foods to unknown and especially initially less tasty but healthier alternatives) or specific barriers (e.g., pills too big to swallow for children; costs or accessibility of healthier food choices or specific nutritional supplements).

Data on these variables will be extracted from all studies eligible for the quantitative analyses. We will also approach the authors of eligible studies to inquire about implementation barriers and potential solutions both in relation to the specific study and in general regarding the intervention they used in the given study. This will help us utilize expert knowledge in this regard accumulated to date, not just up to the point of publication.

Finally, we will also search information on implementation barriers and facilitators on websites of relevant governmental and international agencies, professional organizations, and in regular scientific articles through Google Scholar.

#### 3.3.15 Summary of findings and assessment of the certainty of the evidence

We will consider to formally assess the certainty of the evidence using the Grading of Recommendations, Assessment, Development and Evaluation (GRADE) approach (Guyatt et al., 2008) if time and resources permit, which cannot be guaranteed due to the tight timeline agreed upon with the funder. We do not plan to include a formal Summary of findings table.

## CONTRIBUTIONS OF AUTHORS

- Content: Barna Konkolÿ Thege, Olivia Choy
- Systematic review methods: Barna Konkolÿ Thege, Jamie Hartmann‐Boyce, Olivia Choy
- Statistical analysis: Barna Konkolÿ Thege, Jamie Hartmann‐Boyce
- Information retrieval: Eden Kinzel

## DECLARATIONS OF INTEREST

Olivia Choy was involved in the publication of two randomized controlled trials of omega‐3/vitamin D supplementation on young people who have offended and children with externalizing behavior disorder. The other authors of this systematic review and meta‐analysis have no conflicts of interest.

## PRELIMINARY TIMEFRAME

Approximate date for submission of the systematic review will be no longer than 1 year after protocol approval.

## PLANS FOR UPDATING THIS REVIEW

Once completed, this systematic review may be updated every five years by the senior author (B.K.T.) depending on resource availability.

## Supporting information

Supporting information.

## References

[cl21400-bib-0002] Achenbach, T. M. , & Ruffle, T. M. (2000). The child behavior checklist and related forms for assessing behavioral/emotional problems and competencies. Pediatrics in Review, 21(8), 265–271. 10.1542/pir.21-8-265 10922023

[cl21400-bib-0003] Adan, R. A. H. , van der Beek, E. M. , Buitelaar, J. K , Cryan, J. F , Hebebrand, J. , Higgs, S. , Schellekens, H. , & Dickson, S. L. (2019). Nutritional psychiatry: Towards improving mental health by what you eat. European Neuropsychopharmacology, 29(12), 1321–1332. 10.1016/j.euroneuro.2019.10.011 31735529

[cl21400-bib-0004] Adesanya, D. O. , Johnson, J. , & Galanter, C. A. (2021). Assessing and treating aggression in children and adolescents. Pediatric Medicine, 5, 18.

[cl21400-bib-0005] Aman, M. G. , Singh, N. N. , Stewart, A. W. , & Field, C. J. (1985). The Aberrant Behavior Checklist: A behavior rating scale for the assessment of treatment effects. American Journal of Mental Deficiency, 89(5), 485–491. 3993694

[cl21400-bib-0006] Arain, M. , Haque, M. , Johal, L. , Mathur, P. , Nel, W. , Rais, A. , Sandhu, R. , & Sharma, S. (2013). Maturation of the adolescent brain. Neuropsychiatric Disease and Treatment, 9, 449–461. 10.2147/NDT.S39776 23579318 PMC3621648

[cl21400-bib-0007] Bailey, R. L. , Gahche, J. J. , Lentino, C. V. , Dwyer, J. T. , Engel, J. S. , Thomas, P. R. , Betz, J. M. , Sempos, C. T. , & Picciano, M. F. (2010). Dietary supplement use in the United States, 2003–2006. Journal of Nutrition, 141(2), 261–266. 10.3945/jn.110.133025 21178089 PMC3021445

[cl21400-bib-0008] Barr, A. , & Smith, A. A. (2023). Fighting crime in the cradle. The Effects of Early Childhood Access to Nutritional Assistance, 58(1), 43–73. 10.3368/jhr.58.3.0619-10276R2

[cl21400-bib-0009] Barzman Drew, H , & Findling Robert, L. (2008). Pharmacological treatment of pathologic aggression in children. International Review of Psychiatry, 20(2), 151–157. 10.1080/09540260801887819 18386205

[cl21400-bib-0010] Benton, D. (2007). The impact of diet on anti‐social, violent and criminal behaviour. Neuroscience & Biobehavioral Reviews, 31(5), 752–774. 10.1016/j.neubiorev.2007.02.002 17433442

[cl21400-bib-0011] Bradshaw C. P. , Schaeffer C. M. , Petras H. , Ialongo N . Predicting negative life outcomes from early aggressive–disruptive behavior trajectories: Gender differences in maladaptation across life domains. Journal of Youth and Adolescence 2010;39(8):953–966. 10.1007/s10964-009-9442-8 19688587

[cl21400-bib-0012] Buss, A. H. , & Perry, M. (1992). The Aggression Questionnaire. Journal of Personality and Social Psychology, 63(3), 452–459. 10.1037//0022-3514.63.3.452 1403624

[cl21400-bib-0013] Chan, E. , Rappaport, L. A. , & Kemper, K. J. (2003). Complementary and alternative therapies in childhood attention and hyperactivity problems. Journal of Developmental & Behavioral Pediatrics, 24(1), 4–8. 12584479 10.1097/00004703-200302000-00003

[cl21400-bib-0014] Chorpita, B. F. , Daleiden, E. L. , Ebesutani, C. , Young, J. , Becker, K. D. , Nakamura, B. J. , Phillips, L. , Ward, A. , Lynch, R. , Trent, L. , Smith, R. L. , Okamura, K. , & Starace, N (2011). Evidence‐based treatments for children and adolescents: An updated review of indicators of efficacy and effectiveness. Clinical Psychology: Science and Practice, 18(2), 154–172. 10.1111/j.1468-2850.2011.01247.x

[cl21400-bib-0015] Christian, L. M. , Galley, J. D. , Hade, E. M. , Schoppe‐Sullivan, S. , Claire, K. D. , & Bailey, M. T. (2015). Gut microbiome composition is associated with temperament during early childhood. Brain, Behavior, and Immunity, 45, 118–127. 10.1016/j.bbi.2014.10.018 25449582 PMC4342262

[cl21400-bib-0016] Cleverley, K. , Szatmari, P. , Vaillancourt, T. , Boyle, M. , & Lipman, E. (2012). Developmental trajectories of physical and indirect aggression from late childhood to adolescence: Sex differences and outcomes in emerging adulthood. Journal of the American Academy of Child & Adolescent Psychiatry, 51(10), 1037–1051. 10.1016/j.jaac.2012.07.010 23021479

[cl21400-bib-0017] Dinan, T. G. , Stilling, R. M. , Stanton, C. , & Cryan, J. F (2015). Collective unconscious: How gut microbes shape human behavior. Journal of Psychiatric Research, 63, 1–9. 10.1016/j.jpsychires.2015.02.021 25772005

[cl21400-bib-0018] Dinan T . (2023). Nutritional psychiatry: A primer for clinicians. 10.1017/9781009299862

[cl21400-bib-0019] Ebesutani, C. , Bernstein, A. , Martinez, J. I. , Chorpita, B. F. , & Weisz, J. R. (2011). The Youth Self Report: Applicability and validity across younger and older youths. Journal of Clinical Child & Adolescent Psychology, 40(2), 338–346. 10.1080/15374416.2011.546041 21391029

[cl21400-bib-0020] Eldridge, S. , Campbell, M. K. , Campbell, M. J. , Drahota, A. K. , Giraudeau, B. , Reeves, B. C. , Siegfried, N. , & Higgins, J. P. T. (2021). *Revised Cochrane risk of bias tool for randomized trials (RoB 2). Additional considerations for cluster‐randomized trials (RoB 2 CRT)*.

[cl21400-bib-0021] Eves, A. , & Gesch, B. (2003). Food provision and the nutritional implications of food choices made by young adult males, in a young offenders’ institution. Journal of Human Nutrition and Dietetics, 16(3), 167–179. 10.1046/j.1365-277X.2003.00438.x 12753110

[cl21400-bib-0022] Food and Drug Administration . (2016). *Food labeling: Revision of the nutrition and supplement facts labels*. 27236870

[cl21400-bib-0023] Ford, J. D. , Chapman, J. , Connor, D. F. , & Cruise, K. R. (2012). Complex trauma and aggression in secure juvenile justice settings. Criminal Justice and Behavior, 39(6), 694–724. 10.1177/0093854812436957

[cl21400-bib-0024] Fossati, A. , Raine, A. , Borroni, S. , Bizzozero, A. , Volpi, E. , Santalucia, I. , & Maffei, C. (2009). A cross‐cultural study of the psychometric properties of the Reactive–Proactive Aggression Questionnaire among Italian nonclinical adolescents. Psychological Assessment, 21(1), 131–135. 10.1037/a0014743 19290773

[cl21400-bib-0025] Gajos, J. M. , & Beaver, K. M. (2016). The effect of omega‐3 fatty acids on aggression: A meta‐analysis. Neuroscience and Biobehavioral Review, 69, 147–158. 10.1016/j.neubiorev.2016.07.017 27450580

[cl21400-bib-0026] Galler, J. R. , Bryce, C. P. , Waber, D. P. , Hock, R. S. , Harrison, R. , Eaglesfield, G. D. , & Fitzmaurice, G. (2012). Infant malnutrition predicts conduct problems in adolescents. Nutritional Neuroscience, 15(4), 186–192. 10.1179/1476830512Y.0000000012 22584048 PMC3782078

[cl21400-bib-0027] Gardiner, P. , Buettner, C. , Davis, R. B. , Phillips, R. S. , & Kemper, K. J. (2008). Factors and common conditions associated with adolescent dietary supplement use: An analysis of the National Health and Nutrition Examination Survey (NHANES). BMC Complementary and Alternative Medicine, 8(1):9. 10.1186/1472-6882-8-9 18377653 PMC2330139

[cl21400-bib-0028] Goodman, R. (2001). Psychometric properties of the Strengths and Difficulties Questionnaire. Journal of the American Academy of Child & Adolescent Psychiatry, 40(11), 1337–1345. 10.1097/00004583-200111000-00015 11699809

[cl21400-bib-0029] Gorman, D. A. , Gardner, D. M. , Murphy, A. L. , Feldman, M. , Bélanger, S. A. , Steele, M. M. , Boylan, K. , Cochrane‐Brink, K. , Goldade, R. , Soper, P. R. , Ustina, J. , & Pringsheim, T. (2015). Canadian guidelines on pharmacotherapy for disruptive and aggressive behaviour in children and adolescents with attention‐deficit hyperactivity disorder, oppositional defiant disorder, or conduct disorder. Canadian Journal of Psychiatry, 60(2), 62–76. 10.1177/070674371506000204 25886657 PMC4344948

[cl21400-bib-0030] Guyatt, G. H. , Oxman, A. D. , Vist, G. E. , Kunz, R. , Falck‐Ytter, Y. , Alonso‐Coello, P. , & Schünemann, H. J (2008). GRADE: An emerging consensus on rating quality of evidence and strength of recommendations. BMJ, 336(7650), 924–926. 10.1136/bmj.39489.470347.AD 18436948 PMC2335261

[cl21400-bib-0031] Hannan‐Jones, M , & Capra, S. (2016). Prevalence of diet‐related risk factors for chronic disease in male prisoners in a high secure prison. European Journal of Clinical Nutrition, 70(2), 212–216. 10.1038/ejcn.2015.100 26081491

[cl21400-bib-0032] Hedges, L. V. (2007). Effect sizes in cluster‐randomized designs. Journal of Educational and Behavioral Statistics, 32(4), 341–370. 10.3102/1076998606298043

[cl21400-bib-0033] Hibbeln, J. R. , & Gow, R. V. (2014). The potential for military diets to reduce depression, suicide, and impulsive aggression: A review of current evidence for omega‐3 and omega‐6 fatty acids. Military Medicine, 179(Suppl. 11), 117–128. 10.7205/milmed-d-14-00153 25373095

[cl21400-bib-0034] Higgins, J. P. T. , Thompson, S. G , Deeks, J. J , & Altman, D. G. (2003). Measuring inconsistency in meta‐analyses. BMJ, 327, 557–560. 10.1136/bmj.327.7414.557 12958120 PMC192859

[cl21400-bib-0035] Higgins J. P. T. , Savović J. , Page M. J. , Sterne J. A. C . (2019). *Revised Cochrane risk‐of‐bias tool for randomized trials (RoB 2)*.

[cl21400-bib-0036] Higgins, J. P. T. , Li, T. , & Sterne, J. (2021). *Revised Cochrane risk of bias tool for randomized trials (RoB 2): Additional considerations for crossover trials Cochrane Methods*.

[cl21400-bib-0037] Huesmann, L. R. , Dubow, E. F. , & Paul, B. (2009). Continuity of aggression from childhood to early adulthood as a predictor of life outcomes: Implications for the adolescent‐limited and life‐course‐persistent models. Aggressive Behavior, 35(2), 136–149. 10.1002/ab.20300 19189380 PMC4513937

[cl21400-bib-0038] Jackson, D. B. , Newsome, J. , Vaughn, M. G. , & Johnson, K. R. (2018). Considering the role of food insecurity in low self‐control and early delinquency. Journal of Criminal Justice, 56, 127–139. 10.1016/j.jcrimjus.2017.07.002

[cl21400-bib-0039] Jacobs, E. T. , & Mullany, C. J. (2015). Vitamin D deficiency and inadequacy in a correctional population. Nutrition, 31(5), 659–663. 10.1016/j.nut.2014.10.010 25837209

[cl21400-bib-0040] Kaplan, B. J. , Isaranuwatchai, W. , & Hoch, J. S. (2017). Hospitalization cost of conventional psychiatric care compared to broad‐spectrum micronutrient treatment: literature review and case study of adult psychosis. International Journal of Mental Health Systems, 11(1), 14. 10.1186/s13033-017-0122-x 28163777 PMC5282873

[cl21400-bib-0041] Kaplan B. J , & Rucklidge J. J. (2021). *The better brain: Overcome anxiety, combat depression, and reduce ADHD and stress with nutrition*. Houghton Mifflin Harcourt.

[cl21400-bib-0042] Kugley, S. , Wade, A. , Thomas, J. , Mahood, Q. , Klint, J. A.‐M. , Hammerstrøm, K. , & Sathe, N. (2017). Searching for studies: a guide to information retrieval for Campbell systematic reviews. Campbell Systematic Reviews, 13(1), 1–73. 10.4073/cmg.2016.1

[cl21400-bib-0043] Lee, A. H. , & DiGiuseppe, R. (2018). Anger and aggression treatments: a review of meta‐analyses. Current Opinion in Psychology, 19, 65–74. 10.1016/j.copsyc.2017.04.004 29279226

[cl21400-bib-0044] Liu, J. , Raine, A. , Venables, P. H. , & Mednick, S. A. (2004). Malnutrition at age 3 years and externalizing behavior problems at ages 8, 11, and 17 years. American Journal of Psychiatry, 161(11), 2005–2013. 10.1176/appi.ajp.161.11.2005 15514400 PMC1570126

[cl21400-bib-0045] López‐López, J. A , Page, M. J. , Lipsey, M. W. , & Higgins, J. P. T. (2018). Dealing with effect size multiplicity in systematic reviews and meta‐analyses. Research Synthesis Methods, 9(3), 336–351. 10.1002/jrsm.1310 29971966

[cl21400-bib-0046] Magalotti, S. R. , Neudecker, M. , Zaraa, S. G. , & McVoy, M. K. (2019). Understanding chronic aggression and its treatment in children and adolescents. Current Psychiatry Reports, 21(12), 123. 10.1007/s11920-019-1105-1 31741142

[cl21400-bib-0047] Martínez‐López, E. , Pérez‐Guerrero Edsaúl, E. , Torres‐Carrillo, N. M. , López‐Quintero, A. , Betancourt‐Núñez, A. , & Gutiérrez‐Hurtado, I. A. (2022). Methodological aspects in randomized clinical trials of nutritional interventions. Nutrients, 14(12):2365. 10.3390/nu14122365 35745096 PMC9227614

[cl21400-bib-0048] Marx, W. , Moseley, G. , Berk, M. , & Jacka, F. (2017). Nutritional psychiatry: the present state of the evidence. Proceedings of the Nutrition Society, 76(4), 427–436. 10.1017/S0029665117002026 28942748

[cl21400-bib-0049] McGowan, J. , Sampson, M. , Salzwedel, D. M. , Cogo, E. , Foerster, V. , & Lefebvre, C. (2016). PRESS peer review of electronic search strategies: 2015 guideline statement. Journal of Clinical Epidemiology, 75, 40–46. 10.1016/j.jclinepi.2016.01.021 27005575

[cl21400-bib-0050] Methods Coordinating Group of the Campbell Collaboration . (2019). *Methodological expectations of Campbell Collaboration intervention reviews: Reporting standards*. 10.4073/cpg.2016.3

[cl21400-bib-0051] Muth, A.‐K. , & Park, S. Q. (2021). The impact of dietary macronutrient intake on cognitive function and the brain. Clinical Nutrition, 40(6), 3999–4010. 10.1016/j.clnu.2021.04.043 34139473

[cl21400-bib-0052] Okada, L. M. , Marques, E. S. , Levy, R. B. , Peres, M. F. T. , & Azeredo, C. M . Association between dietary patterns and bullying among adolescents in Sao Paulo—Brazil. *International Journal of Offender Therapy and Comparative Criminology*, 68(4), 299–316. 10.1177/0306624X221095017 35535611

[cl21400-bib-0053] Patrick, R. P. , & Ames, B. N. (2015). Vitamin D and the omega‐3 fatty acids control serotonin synthesis and action, part 2: Relevance for ADHD, bipolar disorder, schizophrenia, and impulsive behavior. The FASEB Journal, 29(6), 2207–2222. 10.1096/fj.14-268342 25713056

[cl21400-bib-0054] Pietro, M. , Valentina, L. , Azzurra, M. , Laura, R. , & Furio, L. (2018). Chapter 6: Parent training interventions for children and adolescents with aggressive behavioral problems. In L. Benedetto , & M. Ingrassia (Eds.), Parenting. IntechOpen. 10.5772/intechopen.73541

[cl21400-bib-0055] Pisano, S. , & Masi, G. (2020). Recommendations for the pharmacological management of irritability and aggression in conduct disorder patients. Expert Opinion on Pharmacotherapy, 21(1), 5–7. 10.1080/14656566.2019.1685498 31663786

[cl21400-bib-0056] Qamar, R. , Wang, S. M. , Qureshi, F. M. , LaChance, L. , Kolla, N. J. , & Konkolÿ, T. B. (2023). Nutritional supplementation in the management of childhood/youth aggression: A systematic review. Aggression and Violent Behavior, 71, 101841. 10.1016/j.avb.2023.101841

[cl21400-bib-0057] Qato, D. M. , Alexander, G. C. , Guadamuz, J. S. , & Lindau, S. T. (2018). Prevalence of dietary supplement use in US children and adolescents, 2003–2014. JAMA Pediatrics, 172(8), 780–782. 10.1001/jamapediatrics.2018.1008 29913013 PMC6142922

[cl21400-bib-0058] Qureshi, F. M. , Kunaratnam, N. , Kolla, N. J. , & Konkolÿ, T. B. (2021). Nutritional supplementation in the treatment of violent and aggressive behavior: A systematic review. Aggressive Behavior, 47(3), 296–309. 10.1002/ab.21953 33580517

[cl21400-bib-0059] Raine, A. , Dodge, K. , Loeber, R. , Gatzke‐Kopp, L. , Lynam, D. , Reynolds, C. , Stouthamer‐Loeber, M. , & Liu, J. (2006). The Reactive–Proactive Aggression Questionnaire: Differential correlates of reactive and proactive aggression in adolescent boys. Aggressive Behavior, 32(2), 159–171. 10.1002/ab.20115 20798781 PMC2927832

[cl21400-bib-0060] Roberts, M. , Tolar‐Peterson, T. , Reynolds, A. , Wall, C. , Reeder, N. , & Rico, M. G. (2022). The effects of nutritional interventions on the cognitive development of preschool‐age children: A systematic review. Nutrients, 14(3):532. 10.3390/nu14030532 35276891 PMC8839299

[cl21400-bib-0061] Robinson, S. L. , Mora‐Plazas, M. , Oliveros, H. , Marin, C. , Lozoff, B. , & Villamor, E. (2021). Dietary patterns in middle childhood and behavior problems in adolescence. European Journal of Clinical Nutrition, 75(12), 1809–1818. 10.1038/s41430-021-00888-4 33674775

[cl21400-bib-0062] Rosato, N. S. , Correll, C. U. , Pappadopulos, E. , Chait, A. , Crystal, S. , & Jensen, P. S. (2012). Treatment of maladaptive aggression in youth: CERT guidelines II. Treatments and ongoing management. Pediatrics, 129(6), 1577–1586. 10.1542/peds.2010-1361 22641763

[cl21400-bib-0063] Rosell, D. R. , & Siever, L. J. (2015). The neurobiology of aggression and violence. CNS Spectrums, 20(3), 254–279. 10.1017/S109285291500019X 25936249

[cl21400-bib-0064] Rucklidge, J. J. , & Kaplan, B. J. (2013). Broad‐spectrum micronutrient formulas for the treatment of psychiatric symptoms: A systematic review. Expert Review of Neurotherapeutics, 13(1), 49–73. 10.1586/ern.12.143 23253391

[cl21400-bib-0065] Rucklidge, J. J. , Kaplan, B. J. , & Mulder, R. T. (2015). What if nutrients could treat mental illness? Australian & New Zealand Journal of Psychiatry, 49(5), 407–408. 10.1177/0004867414565482 25586756

[cl21400-bib-0066] Rucklidge, J. J. , Johnstone, J. M. , & Kaplan, B. J. (2021). Nutrition provides the essential foundation for optimizing mental health. Evidence‐Based Practice in Child and Adolescent Mental Health, 6(1), 131–154. 10.1080/23794925.2021.1875342

[cl21400-bib-0067] Sarris, J. , Logan, A. C. , Akbaraly, T. N. , Amminger, G. P. , Balanzá‐Martínez, V. , Freeman, M. P. , Hibbeln, J. , Matsuoka, Y. , Mischoulon, D. , Mizoue, T. , Nanri, A. , Nishi, D. , Ramsey, D. , Rucklidge, J. J , Sanchez‐Villegas, A. , Scholey, A. , Su, K.‐P. , & Jacka, F. N. (2015). Nutritional medicine as mainstream in psychiatry. Lancet Psychiatry, 2(3), 271–274. 10.1016/S2215-0366(14)00051-0 26359904

[cl21400-bib-0068] Siever, L. J. (2008). Neurobiology of aggression and violence. American Journal of Psychiatry, 165(4), 429–442. 10.1176/appi.ajp.2008.07111774 18346997 PMC4176893

[cl21400-bib-0069] Smoyer, A. B. (2019). Food in correctional facilities: A scoping review. Appetite, 141, 104312. 10.1016/j.appet.2019.06.004 31202918

[cl21400-bib-0070] Soltysova, M. , Tomova, A. , & Ostatnikova, D. (2022). Gut microbiota profiles in children and adolescents with psychiatric disorders. Microorganisms, 10(10), 2009. 10.3390/microorganisms10102009 36296284 PMC9608804

[cl21400-bib-0071] Sterne, J. A. C. , Sutton, A. J. , Ioannidis, J. P. A. , Terrin, N. , Jones, D. R. , Lau, J. , Carpenter, J. , Rücker, G. , Harbord, R. M. , Schmid, C. H. , Tetzlaff, J. , Deeks, J. J. , Peters, J. , Macaskill, P. , Schwarzer, G. , Duval, S. , Altman, D. G. , Moher, D. , & Higgins, J. P. T. (2011). Recommendations for examining and interpreting funnel plot asymmetry in meta‐analyses of randomised controlled trials. BMJ, 343, d4002. 10.1136/bmj.d4002 21784880

[cl21400-bib-0072] Sterne, J. A. C. , Hernán, M. A. , Reeves, B. C. , Savović, J. , Berkman, N. D. , Viswanathan, M. , Henry, D. , Altman, D. G. , Ansari, M. T. , Boutron, I. , Carpenter, J. R. , Chan, A.‐W. , Churchill, R. , Deeks, J. J. , Hróbjartsson, A. , Kirkham, J. , Jüni, P. , Loke, Y. K. , Pigott, T. D. , … Higgins, J. P. T (2016). ROBINS‐I: A tool for assessing risk of bias in non‐randomised studies of interventions. BMJ, 355, i4919. 10.1136/bmj.i4919 27733354 PMC5062054

[cl21400-bib-0073] Tcherni‐Buzzeo M . Dietary interventions, the gut microbiome, and aggressive behavior: Review of research evidence and potential next steps. Aggressive Behavior 2023;49(1), 15–32. 10.1002/ab.22050 35997420

[cl21400-bib-0074] Trapp, G. S. A. , Allen, K. L. , Black, L. J. , Ambrosini, G. L. , Jacoby, P. , Byrne, S. , Martin, K. E. , & Oddy, W. H. (2016). A prospective investigation of dietary patterns and internalizing and externalizing mental health problems in adolescents. Food Science & Nutrition, 4(6), 888–896. 10.1002/fsn3.355 27826439 PMC5090653

[cl21400-bib-0075] Tripathy, S. , Negi, S. , Kumar, D. , & Shamim, M. A. (2023). Prevalence of Vitamin‐D deficiency and insufficiency among prisoners across the globe: A systematic review and meta‐analysis. Journal of Forensic and Legal Medicine, 97, 102549. 10.1016/j.jflm.2023.102549 37348178

[cl21400-bib-0076] Vatanparast, H. , Adolphe, J. L , & Whiting, S. J. (2010). Socio‐economic status and vitamin/mineral supplement use in Canada. Health Reports, 21(4), 19–25. 21269008

[cl21400-bib-0077] White, H. (2018). Theory‐based systematic reviews. Journal of Development Effectiveness, 10(1), 17–38. 10.1080/19439342.2018.1439078

[cl21400-bib-0078] Wilson, D. B. (2001). *Practical meta‐analysis effect size calculator* [Online calculator].

[cl21400-bib-0079] Woodall, J. (2016). A critical examination of the health promoting prison two decades on. Critical Public Health, 26(5), 615–621. 10.1080/09581596.2016.1156649

[cl21400-bib-0080] World Health Organization . (2003). *Adherence to long‐term therapies. Evidence for action*.

