# Supplementary material for: PROTOCOL: The efficacy of nutritional interventions in reducing childhood/youth aggressive and antisocial behavior: A systematic review and meta‐analysis
Source: Campbell Syst Rev. 2024 Apr 18;20(2):e1400. doi: 10.1002/cl2.1400 (PMC11024636; doi:10.1002/cl2.1400)
Supplement: Supplementary file 1 — Supporting information. [file CL2-20-e1400-s001.docx]

**Appendices**

**Appendix 1. Draft of the Ovid MEDLINE search string to be used during the electronic data base searches**

| # | Searches |
| --- | --- |
| 1 | (aggress* adj5 (behav* or conduct* or disorder* or issue* or problem* or demeanor or act*)).tw,kf. |
| 2 | (aggressiveness or aggression*).tw,kf. |
| 3 | (hetero adj3 aggress*).tw,kf. |
| 4 | heteroaggress*.kf,tw. |
| 5 | (extraggress* or hyperaggress* or microaggress*).kf,tw. |
| 6 | externaliz*.kf,tw. |
| 7 | ((oppositional* adj4 (defy or defian*)) or ((defy or defian*) adj4 disorder*) or (oppositional* adj4 disorder*)).kf,tw. |
| 8 | (conduct adj3 (disorder* or issue* or problem*)).kf,tw. |
| 9 | (anti-social* or antisocial* or sadis* or sociopath* or psychopath*).kf,tw. |
| 10 | (deliquen* or disobey* or disobedien* or shoplift* or shop-lift* or vandal* or arson* or rob or robber* or burglar* or assault* or murder* or homicide* or stalk* or incivility or kidnap* or manslaughter* or harass* or recidiv* or reoffend* or offen*).kf,tw. |
| 11 | violen*.kf,tw. |
| 12 | (rape or raped or rapes or raping or rapist* or molest*).kf,tw. |
| 13 | (sex* adj3 (nonconsen* or non-consen* or violat* or dominan* or coerc* or abus*)).kf,tw. |
| 14 | (fight* or fought or combative*).kf,tw. |
| 15 | (((explos* or explod*) adj4 disorder*) or (intermittent* adj4 (explod* or explos*))).kf,tw. |
| 16 | (bully* or bulli* or cyberbull*).kf,tw. |
| 17 | aggression/ |
| 18 | problem behavior/ or cyberbullying/ or bullying/ |
| 19 | Conduct Disorder/ or "Disruptive, Impulse Control, and Conduct Disorders"/ or Antisocial Personality Disorder/ |
| 20 | homicide/ or recidivism/ or sex offenses/ or rape/ or theft/ or violence/ or domestic violence/ or gun violence/ or intimate partner violence/ or physical abuse/ or workplace violence/ or Harassment, Non-Sexual/ or Sexual Harassment/ or firesetting behavior/ or emotional abuse/ or incivility/ or juvenile delinquency/ or stalking/ or criminal behavior/ |
| 21 | 1 or 2 or 3 or 4 or 5 or 6 or 7 or 8 or 9 or 10 or 11 or 12 or 13 or 14 or 15 or 16 or 17 or 18 or 19 or 20 |
| 22 | (vitamin* or multivitamin*).kf,tw. |
| 23 | (nutri* or macronutri* or micronutri*).kf,tw. |
| 24 | (nutraceutic* or nutrapharm* or nutra-pharm*).kf,tw. |
| 25 | mineral*.kf,tw. |
| 26 | phytoceutic*.kf,tw. |
| 27 | (food or foods or feed or feeds or feeding or fed).kf,tw. |
| 28 | (diet or diets or dietary).kf,tw. |
| 29 | (vegetable* or fruit or fruits or meat or meats or pork or beef or chicken or poultry or venison or veal or shellfish* or fish or (corn adj3 syrup*) or dairy or milk or cheese* or wheat or nondairy or juice or juices or smoothie or smoothies or soda or sodas or coffee* or tea or teas or egg or eggs or water or dessert* or tofu or sugary or plant-base* or plantbase* or vegetarian* or vegan* or keto or ketogenic* or pescetarian* or paleo* or flexitarian* or carnivore* or raw or gluten free or mediterranean or (intermit* adj3 fast*) or prebiotic or prebiotics or pre-biotic or pre-biotics or probiotic or probiotics or pro-biotic or pro-biotics or sweeten*).kf,tw. |
| 30 | ((supplement or supplementing or supplemented or supplements) adj4 (eat* or ate or consum* or ingest* or drink* or drank or beverage* or administer* or give* or giving or gave or provid* or take* or taking or took)).kf,tw. |
| 31 | dh.fs. |
| 32 | ((5htp or 5-htp or (st adj3 john* adj3 wort*) or additive* or amino acid* or antioxidant* or anti-oxidant* or Arachidonic* or ascorb* or Biotin or Boric or Boron or Caffein* or Calciferol or Calcitriol or Calcium or Camphor or carb or carbohydrate* or carbs or Carnitin* or Cassava or Cesium or Chamomile or Chlorid* or chlorin* or Cholecalciferol or Choline or Chromium or Cobalamin or Cobalt or Cobamid* or coenzyme q10 or Copper or Creatin* or dehydroascorb* or Docosahexaeno* or Docosapentaeno* or eicosapentaeno* or Eicosatetraeno* or Eicosatrieno* or Ergocalciferol* or fat or fats or fatty or fiber or fibre or Flax* or Fluorid* or Fluorin* or Folacin or Folate or Folic acid* or Garlic or Ginger or Ginkgo or ginseng or Glucosamine or Glutamine or grain or grains or Histidine or Hydroxocobalamin* or Hydroxymethylbutyrate or hydroxytryptophan or Inositol or Iodine or Iron or Isoleucine or Jimson Weed or Khat or Kratom or legume* or Leucine or Licorice or Linoleic* or linolen* or liquorice or Lysine or Magnesium or Manganese or melatonin or Methionine or Niacin or Nicotinamide or Nicotinic acid or Nicotinuric acid or nut or nuts or Oat or oats or Oil or Phenylalanine or Phosphate* or Potassium or protein or proteins or Pyridoxine or retinaldehyde* or retinoic or retinoid or retinol or retinyl* or Riboflavin or Selenium or Sodium or Soy or soya or soybean or starch* or sugar* or Sulfur or Taurine or Thiamin* or Threonine or Tin or tocopherol* or Tocotrienol* or transfat* or triglycerid* or tri-glycerid* or Tryptophan or ubiquinon* or Valine or Whey or Yerba Mate or Yogurt* or yoghurt* or Zinc*) adj4 (eat* or ate or consum* or ingest* or drink* or drank or beverage* or supplement or supplements or pill or pills or tablet* or capsule*)).kf,tw. |
| 33  34  35  36  37  38  39 | ((pufa or pufas or mufa or mufas or (omega adj3 ("3" or "6" or "9" or "12" or oil* or fat or acid*))).kf,tw.  ((herb* or eastern or Chinese or plant* or traditional or ancient or alternative or complementary or natural) adj4 (medic* or remed* or heal* or drug or drugs) adj4 (eat* or ate or consum* or ingest* or drink* or drank or beverage* or supplement or supplements or pill or pills or tablet* or capsule*)).kf,tw.  ((herb* or plant or plants) adj4 (product* or preparation* or therap* or treat* or extract*) adj4 (eat* or ate or consum* or ingest* or drink* or drank or beverage* or supplement or supplements or pill or pills or tablet* or capsule*)).kf,tw.  ((ethnopharm* or phytotherap* or phytomedicin* or ethnobotan* or ethnomedic* or botanical*) adj4 (eat* or ate or consum* or ingest* or drink* or drank or beverage* or supplement or supplements or pill or pills or tablet* or capsule*)).kf,tw.  ((sport* or electrolyte or soft or carbonat* or fizz* or energy) adj4 (drink or drinks or beverage*)).kf,tw.  ((high or low or mid or moderate or less or more) adj4 (carb* or protein or proteins or fat or fats or fatty)).kf,tw.  ((processed or organic or junk) adj4 (eat* or ate or consum* or ingest* or drink* or drank or beverage*)).kf,tw. |
| 40 | (trace elements/ or 24,25-dihydroxyvitamin d 3/ or 25-hydroxyvitamin d 2/ or calcifediol/ or cholecalciferol/ or cod liver oil/ or beta carotene/ or acetylcarnitine/ or biotin/ or folic acid/ or inositol/ or niacin/ or pyridoxal/ or pyridoxamine/ or pyridoxine/ or riboflavin/ or thiamine/ or synbiotics/ or food preservatives/ or plant preparations/ or plant extracts/ or flower essences/ or curare/ or drugs, chinese herbal/ or lecithins/ or plant oils/ or eucalyptus oil/ or rapeseed oil/ or castor oil/ or clove oil/ or linseed oil/ or palm oil/ or rice bran oil/ or sunflower oil/ or "tea tree oil"/ or Phytotherapy/ or Phytochemicals/ or lipids/ or fatty acids/ or eicosanoic acids/ or fatty acids, unsaturated/ or eicosanoids/ or arachidonic acids/ or arachidonic acid/ or complementary therapies/ or hydroxyeicosatetraenoic acids/ or 12-hydroxy-5,8,10,14-eicosatetraenoic acid/ or eicosapentaenoic acid/ or 5,8,11,14-eicosatetraynoic acid/ or 8,11,14-eicosatrienoic acid/ or fatty acids, essential/ or linoleic acids/ or linoleic acid/ or linolenic acids/ or alpha-linolenic acid/ or gamma-linolenic acid/ or fatty acids, monounsaturated/ or capsaicin/ or erucic acids/ or oleic acids/ or oleic acid/ or undecylenic acids/ or fatty acids, omega-3/ or docosahexaenoic acids/ or fatty acids, omega-6/ or linoleic acids, conjugated/ or sorbic acid/ or trans fatty acids/ or heptanoic acids/ or palmitic acids/ or palmitates/ or palmitic acid/ or stearic acids/ or stearates/ or triglycerides/ or "amino acids, peptides, and proteins"/ or amino acids/ or histidine/ or isoleucine/ or leucine/ or lysine/ or methionine/ or phenylalanine/ or threonine/ or tryptophan/ or valine/ or proteins/ or antioxidants/ or ascorbic acid/ or ergothioneine/ or grape seed extract/ or lycopene/ or melatonin/ or quercetin/ or resveratrol/ or silymarin/ or thioctic acid/ or zeta carotene/) and (eat* or ate or consum* or ingest* or drink* or drank or beverage*).kf,tw. |
| 41 | micronutrients/ or vitamins/ or vitamin a/ or vitamin d/ or vitamin e/ or vitamin k/ or vitamin k 1/ or vitamin k 2/ or vitamin k 3/ or vitamin u/ or provitamins/ or vitamin b complex/ or vitamin b 12/ or vitamin b 6/ or "diet, food, and nutrition"/ or beverages/ or artificially sweetened beverages/ or carbonated beverages/ or carbonated water/ or drinking water/ or energy drinks/ or fermented beverages/ or buttermilk/ or kefir/ or kombucha tea/ or koumiss/ or "fruit and vegetable juices"/ or milk/ or cultured milk products/ or whey/ or milk substitutes/ or soy milk/ or sugar-sweetened beverages/ or tea/ or teas, herbal/ or teas, medicinal/ or fermented foods/ or soy foods/ or food/ or candy/ or chocolate/ or spices/ or edible grain/ or whole grains/ or dairy products/ or whey proteins/ or dietary carbohydrates/ or dietary sugars/ or dietary sucrose/ or high fructose corn syrup/ or dietary fats/ or dietary fats, unsaturated/ or corn oil/ or cottonseed oil/ or olive oil/ or safflower oil/ or sesame oil/ or soybean oil/ or dietary fiber/ or prebiotics/ or dietary proteins/ or animal proteins, dietary/ or egg proteins, dietary/ or meat proteins/ or fish proteins, dietary/ or poultry proteins/ or shellfish proteins/ or milk proteins/ or plant proteins, dietary/ or fruit proteins/ or grain proteins/ or nut proteins/ or pea proteins/ or soybean proteins/ or dietary supplements/ or probiotics/ or yeast, dried/ or eggs/ or egg white/ or egg yolk/ or fast foods/ or flour/ or food ingredients/ or food additives/ or fat substitutes/ or flavoring agents/ or sodium chloride, dietary/ or sweetening agents/ or stevia/ or sucrose/ or sugars/ or xylitol/ or non-nutritive sweeteners/ or nutritive sweeteners/ or food, fortified/ or food, genetically modified/ or food, organic/ or food, preserved/ or frozen foods/ or food, processed/ or foods, specialized/ or food, formulated/ or infant food/ or fruit/ or functional food/ or honey/ or meat/ or meat products/ or poultry/ or poultry products/ or red meat/ or pork meat/ or seafood/ or fish products/ or fish flour/ or shellfish/ or nuts/ or raw foods/ or salads/ or seeds/ or vegetables/ or vegetable products/ or coffee/ or nutrition therapy/ or diet therapy/ or diet, carbohydrate loading/ or diet, carbohydrate-restricted/ or diet, high-protein low-carbohydrate/ or diet, ketogenic/ or diet, fat-restricted/ or diet, gluten-free/ or diet, high-protein/ or diet, mediterranean/ or diet, paleolithic/ or diet, protein-restricted/ or diet, reducing/ or diet, sodium-restricted/ or diet, vegetarian/ or diet, macrobiotic/ or diet, vegan/ or minerals/ |
| 42 | 22 or 23 or 24 or 25 or 26 or 27 or 28 or 29 or 30 or 31 or 32 or 33 or 34 or 35 or 36 or 37 or 38 or 39 or 40 or 41 |
| 43 | young adult/ or infant/ or infant, newborn/ or infant, large for gestational age/ or infant, low birth weight/ or infant, small for gestational age/ or infant, very low birth weight/ or infant, extremely low birth weight/ or infant, postmature/ or infant, premature/ or infant, extremely premature/ or adolescent/ or child/ or child, abandoned/ or child, adopted/ or child, exceptional/ or child, gifted/ or "child of impaired parents"/ or child, foster/ or child, orphaned/ or child, unwanted/ or disabled children/ or child, preschool/ or homeless youth/ or minors/ or adolescent fathers/ or adolescent mothers/ or adolescent, hospitalized/ or adolescent, institutionalized/ or child, hospitalized/ or child, institutionalized/ or Only Child/ or Students/ or Schools/ or Universities/ |
| 44 | (infan* or baby or babies or toddler* or preschool* or kindergar* or child* or girl* or boy* or kid or kids or pediatric* or paediatric* or prepubesc* or preteen* or junior high or juvenile* or youth* or pubescen* or teen* or adolescen* or under-age* or underage* or school* or highschool* or student* or (young adj3 (men or man or woman or women or male* or female* or person* or people* or population* or individual* or adult*)) or emerging adult* or early adult*).kf,tw. |
| 45 | ((college* or universit*) adj5 (women or woman or man or men or people* or person* or attend*)).kf,tw. |
| 46 | (undergrad* or graduate* or post-secondar* or postsecondar*).kf,tw. |
| 47 | 43 or 44 or 45 or 46 |
| 48 | 21 and 41 and 46 |

**Appendix 2. Inclusion and exclusion criteria**

|  | **INCLUSION CRITERIA** | **EXCLUSION CRITERIA** |
| --- | --- | --- |
| Study design | - randomized (or quasi-randomized) controlled trial OR - quasi-experimental (observational cohort design) | - cross-sectional study describing the association between nutritional status / dietary patterns and the selected outcome variables (retain these for enriching discussion) - pre-post design without a control group (retain these for enriching discussion) - systematic review on aggression # nutrition (retain these for enriching discussion) |
| Population / age | - individuals up to the age of 24 regardless of sex/gender - if a study includes participants whose age is > than 24 years old and there is no way to separate those at or under, the study will be included if the mean / median age is 24 years or less. | - animal studies - if the mean (or median) age of the sample is more than 24 years of age, the study will be excluded |
| Population / aggression | - direct or indirect indication of excessive level of aggression at baseline is present (e.g., mental health care utilization for disorders defined by or often correlated with aggression such as ADHD, oppositional defiant disorder, autism, conduct disorder, antisocial personality disorder, being in the criminal justice / correctional system either in a prison or special educational setting, being characterized by above-normal self-rated or observer-rated scale scores measuring aggression etc.) | - no indication of excessive level of aggression either at baseline or intervention end / follow-up |
| Intervention | - includes at least mid-term (minimum of 1 week) dietary modification OR - at least mid-term (minimum of 1 week) nutritional supplementation (either via nutrition-fortified foods or via nutritional supplements / phytoceuticals) | - intervention is medication, which requires medical prescription AND /OR is regulated as a medication (and not as food supplement / natural health product) |
| Comparator | - comparator is non-active (e.g., placebo, treatment as usual) | - comparator is active such as medication, psychosocial intervention (an active comparator study arm should be included though if the intervention group receives the same active comparator in addition to the nutritional intervention) |
| Outcome | - behavioral-level violence / hetero-aggression in real-life settings (including verbal aggression) | - aggression in simulated (e.g., video gaming) settings - aggressive / angry / hostile emotions or thoughts without observable aggressive behavior |
|  | - antisocial behaviors (e.g., disobedience, theft, lying, arson or other property damage as well as other nonviolent offending in combination with OR without aggressive behaviors) | - hyperactivity or other ADHD symptom (e.g., poor attention span, distractibility) in itself without harm done to others |
|  | - violent offending: breaking the law by actual, attempted, or threatened harm directed toward another person (including sexual violence) |  |

**Appendix 3. Draft coding sheet**

| **Variable** | **Response options** | |
| --- | --- | --- |
| General study characteristics | | |
| Authors (publication date) |  | |
| Peer-reviewed | 0 = No  1 = Yes  2 = Unknown | |
| Design | 1 = randomized controlled trial – parallel design  2 = randomized controlled trial – cross-over design (only combined data reported)  3 = randomized controlled trial – block-randomized design  4 = observational cohort design  5 = randomized controlled trial – cross-over design (data reported from phase I) | |
| Sample characteristics | | |
| General characteristics of sample | (e.g., youth prisoners, children with ADHD but not on antidepressants, children with seizure disorders but without taking anticonvulsants etc.) | |
| Setting | (e.g., prison, inpatient psychiatric hospital, school, psychiatric outpatient) | |
| Parallel treatment (e.g., psychotherapy, psychiatric medication) running throughout the trial | 0 = No  1 = Yes; specifically:  2 = Unknown | |
| Default diet / serum nutrient levels |  | |
| Sample size (combined altogether, including treatment and control group) |  | |
| Sex composition | % _male_=  % _female_= | |
| Age | Mean (SD)=  Range=  Median (IR)= | |
| Ethnicity |  | |
| Were ethnicity-stratified analyses conducted regarding outcomes relevant for our purposes? | 0 = No  1 = Yes | |
| Intervention characteristics | | |
| Comparator intervention | (e.g., placebo, capsule of identical appearance containing sunflower oil, treatment as usual) | |
| Intervention type | 1 = dietary manipulation – elimination (e.g.., reduced carbohydrates intake)  2 = dietary manipulation – addition (e.g., consumption of more fish)  3 = dietary manipulation – complete diet change (e.g.., transition to Mediterranean diet)  4 = fortification (e.g., consumption of vitamin D fortified milk)  5 = supplementation (e.g., multivitamin product) | |
| Intervention details | (e.g., 6 mg/kg/day magnesium; drink containing 1,000 mg of omega-3 fatty acids: 300 mg of DHA, 200 mg of EPA, 400 mg of alpha-linolenic acid, and 100 mg of DPA) | |
| Duration of active intervention (in days) |  | |
| Information on implementation (barriers, successful strategies) | (e.g., compliance / drop-out rate for the intervention specifically, cost of dietary change, financial compensation given for participation) | |
| Side effects of intervention |  | |
| Results | | |
| If multiple relevant indicators are reported for the same outcome type,   1. provide rationale for your choice: 2. list ALL non-considered indicators (variable + method of assessment): | | |
| Aggression indicator | (e.g., Reactive Aggression Subscale scores of the Reactive-Proactive Aggression Questionnaire; number of violent rule infractions from prison record) | Can select more than one; if so, select single most relevant result to report for each aggression outcome type (cf. ‘Criteria for determination of independent findings’ in protocol) and extract below data for each of them |
| Aggression outcome type | 1 = hetero-aggression  2 = violent offending  3 = antisocial behaviour  4 = non-violent offending  5= offending: violent & non-violent combined | |
| Aggression outcome rater | 1 = observer-reported  2 = self-reported | |
| Aggression indicator direction | 1 = higher values indicative of more aggression  2 = higher values indicative of less aggression | |
| Attrition rate | Drop-out rate for nutritional intervention:  Drop-out rate from the study altogether (including non-compliance with intervention + non-compliance with assessments): | |
| Score on the ROBINS-I or ROB-2 | 0 = low risk  1 = medium risk  2 = high risk | |
| Summary of findings in relation to the specific outcome addressed here (add as much detail as possible here with alternative metrics if default data for meta-analysis – see below – are not available) | (e.g., no significant difference in aggression between intervention and control group, p-value and effect size not reported; significant time x group interaction at intervention completion: p=0.021, eta squared=0.14) | |
| Treatment group’s size at baseline | (e.g., treatment group comprised of **50** individuals at baseline) | |
| Treatment group’s mean (and SD) at baseline (if outcome is continuous) |  | |
| Number (and %) of cases in treatment group at baseline (if outcome is dichotomous) | (e.g., out of 50 individuals, **25 (50%)** had violent incidents in the baseline assessment period) | |
| Treatment group’s size at intervention-end | (e.g.., treatment group comprised of **40** individuals at intervention-end) | |
| Treatment group’s mean (and SD) at intervention-end (if outcome is continuous) |  | |
| Number (and %) of cases in treatment group at intervention-end (if outcome is dichotomous) | (e.g., out of 40 individuals, **10 (25%)** had violent incidents in the intervention period) | |
| Pre-post effect in treatment group | 1 = improvement (whether statistically significant or not)  2 = deterioration (whether statistically significant or not)  3 = not reported / cannot tell | |
| Control group’s size at baseline | (e.g., Control group comprised of **50** individuals at baseline) | |
| Control group’s mean (and SD) at baseline (if outcome is continuous) |  | |
| Number (and %) of cases in control group at baseline (if outcome is dichotomous) | (e.g., out of 50 individuals, **25 (50%)** had violent incidents in the baseline assessment period) | |
| Control group’s size at intervention-end | (e.g.., control group comprised of **40** individuals at intervention-end) | |
| Control group’s mean (and SD) at intervention-end (if outcome is continuous) |  | |
| Number (and %) of cases in control group at intervention-end (if outcome is dichotomous) | (e.g., out of 40 individuals, **10 (25%)** had violent incidents in the intervention period) | |
| Time between intervention-end and follow-up (in days) |  | |
| Treatment group’s size at follow-up |  | |
| Treatment group’s mean (and SD) at follow-up (if outcome is continuous) |  | |
| Number (and %) of cases in treatment group at follow-up (if outcome is dichotomous) |  | |
| Control group’s size at follow-up |  | |
| Control group’s mean (and SD) at follow-up (if outcome is continuous) |  | |
| Number (and %) of cases in control group at follow-up (if outcome is dichotomous) |  | |
